# Exoskeleton anchoring to tendon cells and muscles in molting isopod crustaceans

**DOI:** 10.3897/zookeys.176.2445

**Published:** 2012-03-20

**Authors:** Nada Žnidaršič, Polona Mrak, Magda Tušek-Žnidarič, Jasna Štrus

**Affiliations:** 1 Department of Biology, Biotechnical Faculty, University of Ljubljana, Večna pot 111, 1000 Ljubljana, Slovenia

**Keywords:** Cuticle, chitin, microtubules, anchoring junctions, extracellular matrix, embryo

## Abstract

Specialized mechanical connection between exoskeleton and underlying muscles in arthropods is a complex network of interconnected matrix constituents, junctions and associated cytoskeletal elements, which provides prominent mechanical attachment of the epidermis to the cuticle and transmits muscle tensions to the exoskeleton. This linkage involves anchoring of the complex extracellular matrix composing the cuticle to the apical membrane of tendon cells and linking of tendon cells to muscles basally. The ultrastructural arhitecture of these attachment complexes during molting is an important issue in relation to integument integrity maintenance in the course of cuticle replacement and in relation to movement ability. The aim of this work was to determine the ultrastructural organization of exoskeleton – muscles attachment complexes in the molting terrestrial isopod crustaceans, in the stage when integumental epithelium is covered by both, the newly forming cuticle and the old detached cuticle. We show that the old exoskeleton is extensively mechanically connected to the underlying epithelium in the regions of muscle attachment sites by massive arrays of fibers in adult premolt *Ligia italica* and in prehatching embryos and premolt marsupial mancas of *Porcellio scaber*. Fibers expand from the tendon cells, traverse the new cuticle and ecdysal space and protrude into the distal layers of the detached cuticle. They likely serve as final anchoring sites before exuviation and may be involved in animal movements in this stage. Tendon cells in the prehatching embryo and in marsupial mancas display a substantial apicobasally oriented transcellular arrays of microtubules, evidently engaged in myotendinous junctions and in apical anchoring of the cuticular matrix. The structural framework of musculoskeletal linkage is basically established in described intramarsupial developmental stages, suggesting its involvement in animal motility within the marsupium.

## Introduction

**Figure 1.**
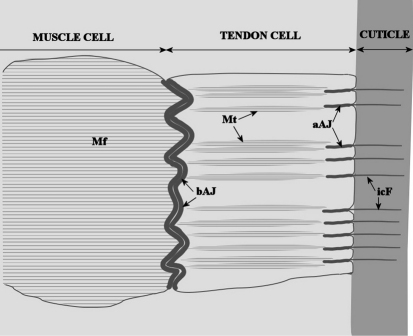
A scheme showing the general architecture of the muscle attachment to the epidermis in arthropods (adapted from Mellon 1992, Lai-Fook and Beaton 1998, Bitsch and Bitsch 2002 and Subramanian et al. 2003). Specialized epithelial cells, named tendon cells, are anchored apically to the cuticle and basally to the muscle cell. **aAJ** apical adherens junction **bAJ** basal adherens junction **icF** intracuticular fibers **Mf** myofilaments **Mt** microtubules.

The arthropod exoskeleton performs diverse functions, including mechanical support, sensing, prevention of desiccation and protection against pathogens and predators. Locomotion of these animals is based on extensive connections between exoskeleton and muscular system. The exoskeleton consists of a complex chitin-protein matrix, secreted by a single-layered epithelium. The chitin-protein matrix is either non-calcified or calcified, as in insects and crustaceans, respectively. Specialized epithelial cells, named tendon cells, are the sites of firm mechanical connections between exoskeleton and underlying tissues (Noble-Nesbitt 1963, Mellon 1992, Lai-Fook and Beaton 1998, Bitsch and Bitsch 2002). Apical membranes of tendon cells are anchored to the matrix of the cuticle and their basal surfaces are attached to muscle cells underneath (Fig. 1). A prominent ultrastructural characteristic of tendon cells are extensive bundles of microtubules, that stretch between the apical cell membrane facing the cuticle and the basal membrane engaged in the myotendinous junction. Thus, the muscle is mechanically linked to epidermis and cuticle by a complex network of cytoskeletal and junctional elements. In addition to their essential role in animal locomotion, muscle cells are expected to play important role at molting, as force-generators for movements that facilitate the exuviation.

The ultrastructure, molecular composition and differentiation of specialized anchoring complexes between exoskeleton, tendon cells and muscle cells were extensively studied in an insect *Drosophila melanogaster*, with emphasis on myotendinous junction characterization (Volk 1999, Brown 2000, Volk 2006, Schweitzer et al. 2010). Apart from *Drosophila*, there is limited data available on the fine structure and constitutive elements of exoskeleton - muscle attachment sites in other arthropods. Studies on the ultrastructural organization of connections between exoskeleton and muscles in the phases of new cuticle formation were performed in some insect species (Lai-Fook 1967, Caveney 1969, Lai-Fook and Beaton 1998) and in two groups of crustaceans: in *Euphausia superba* (Crustacea: Euphausiacea) by Buchholz and Buchholz (1989) and in podocopid ostracods by Yamada and Keyser (2009). Premolt is the unique stage preceding exuviation in which the integumental epithelium is covered by both, the newly forming cuticle apposed to the epithelial cells and the old detached cuticle above the ecdysal space. The key features of this stage in crustaceans are simultaneous disintegration of the old cuticular matrix and new cuticle formation, including secretion and recycling of organic constituents, massive calcium fluxes and modification of epithelial cells shape, size and ultrastructure (Roer and Dillaman 1993, Ziegler 1997, Compere et al. 1998, Štrus and Blejec 2001, Luquet and Marin 2004, Dillaman et al. 2005, Ziegler et al. 2005, Žnidaršič et al. 2010). Exoskeleton renewal inevitably involves the establishment of connections between the new cuticle and muscles. To supplement the knowledge on the ultrastructure of these connections and to address the issues of general principles vs. specializations of their architecture and reorganization related to molting, several species from different environments need to be studied in this respect. The exoskeleton renewal takes place during development and molting in adult specimens. There are no detailed ultrastructural data on exoskeleton anchoring to tendon cells and muscles in molting adults and in marsupial stages of isopod crustaceans. The embryonic development of terrestrial isopod crustaceans and hatching of embryos to marsupial mancas take place inside the female brood pouch (marsupium) and were decribed in *Porcellio scaber* from the view of overall morphology and digestive system morphogenesis, while the issue of cuticle anchoring was not addressed (Štrus et al. 2008, Wolff 2009, Milatovič et al. 2010). Fertilized eggs are released into the brood pouch, where the entire embryonic development takes place. The final phase in the embryonic development is hatching. Newly hatched animals are termed marsupial mancas and they stay inside the brood pouch for up to ten days as described in *Porcellio scaber* females reared in the laboratory (Milatovič et al. 2010).

Here we report new data on the ultrastructural architecture of anchoring complexes comprising exoskeleton, tendon cells and muscles in adult premolt isopod crustaceans, in premolt marsupial mancas and in prehatching embryos. Our study is focused primarily to connections between the complex matrix of the exoskeleton and tendon cells, modified epithelial cells at the sites of muscles attachment. To the best of our knowledge, the exoskeleton anchoring to underlying tissues in embryos and marsupial mancas of crustaceans has not been characterized before and its ultrastructural organization is presented here. Comparative evaluation of the results with respect to the other arthropods, particularly to insect model organism *Drosophila melanogaster*, is presented. The involvement of these anchoring connections in molting and in intramarsupial motility is discussed.

## Methods

Specimens of *Ligia italica* Fabricius, 1798 (Crustacea: Isopoda) were collected at the Piran Bay coast in Slovenia. Animals were inspected for ventral sternal deposits and premolt adult specimens were anaesthetized. The dorsal parts of pereonites were isolated, fixed in 2% paraformaldehyde and 2.5% glutaraldehyde in 0.1 M cacodylate buffer (pH 7.3) and postfixed by 1% OsO_4_. After washing and dehydration in a graded series of ethanol, samples were embedded in an epoxy resin mixture. Semithin sections were stained with Azure II. – Methylene blue. Ultrathin sections were either imaged non-contrasted or contrasted with uranyl acetate and lead citrate.

Specimens of *Porcellio scaber* Latreille, 1804 (Crustacea: Isopoda) prehatching embryos and marsupial mancas were isolated from brood pouches of females maintained in laboratory culture. Determination of intramarsupial developmental stages was performed as described in Milatovič et al. (2010). Isolated embryos and mancas were fixed in 2.5% glutaraldehyde in 0.1 M cacodylate buffer (pH 7.2). Prior to fixation, the vitelline membrane of embryos was carefully perforated with a thin needle. After washing with 0.1 M cacodylate buffer, the samples were postfixed in 1% OsO_4 _for 2 hours, washed again and dehydrated in a graded series of ethanol. Specimens were embedded in Agar 100 resin. Prior to embedding, mancas were perforated with a thin needle for better infiltration of resin. Semithin sections were stained with Azure II. - Methylene blue. Ultrathin sections were contrasted with uranyl acetate and lead citrate.

Light microscopy was performed by AxioImager Z.1 microscope (Zeiss) equiped with an AxioCam HRc camera and Axiovision software. Ultrastructural imaging was performed by CM 100 transmission electron microscope (Phillips) equiped with a BioScan 792 digital camera (Gatan) and Digital Micrograph software.

## Results

### Adult premolt specimens

**Figure 2.**
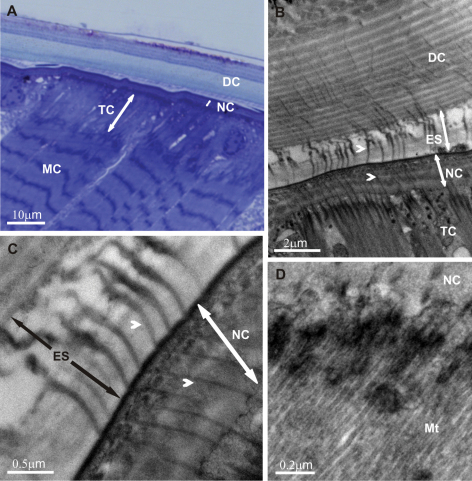
Exoskeleton – muscle attachment in the dorsal parts of pereonites in adult premolt *Ligia italica*. Overview of the muscle attachment in a specimen with a detached and a newly forming cuticle (**2A** a semithin section). The new cuticle is extensively connected to the underlying tendon cells already in the early premolt. Fibrous connections running from the apical region of tendon cells through the new pre-ecdysal cuticle and ecdysal space up to the exocuticle of the detached exoskeleton are evident **(2B, C)**. Parallel arrays of microtubules and apical electron dense plaques are characteristic for tendon cells **(2D)**. **DC** detached cuticle **NC** new cuticle **ES** ecdysal space **MC** muscle cell **TC** tendon cell **Mt** microtubules; arrowheads – fibrous connections.

The ultrastructural architecture of the exoskeleton – muscles attachment regions was analysed in the dorsal parts of pereonites in adult premolt *Ligia italica*. The pre-ecdysial cuticle was tightly connected to the underlying tendon cells and muscles already in the early premolt (Fig. 2). Extensive connections were established between the chitin-protein matrix of the newly forming cuticle and the apical parts of the tendon cells in the early phase of cuticle elaboration. These matrix – cell linkages consisted of numerous fibrous structures. The most intriguing result was that the fibers traversed the entire new cuticle, spanned the whole ecdysal space and protruded deep into the old cuticle (Figs 2B, C). They extended from the tendon cell apex up to the exocuticular layer in the old cuticle. The continuity of these fibers was clearly followed in some sections, revealing a direct mechanical connection of the detached exoskeleton to epithelium in these regions. Fibrous structures were arranged in parallel to one another and in the same direction as microtubules arrays in the tendon cells and myofilaments arrays in the muscle cells. This direction is roughly perpendicular to the body surface. Fibrous connections followed approximately straight lines and did not display any branching or prominent curvatures. General architecture of the preecdysal cuticle at muscle attachment sites was similar to that in the other regions, displaying epicuticular and exocuticular layers and characteristic pattern of chitin-protein fibers arrangement.

Extensive parallel arrays of microtubules inside the tendon cells were aligned in the apical to basal direction (Fig. 2D). A concourse of microtubules towards the cytoplasmic densities at the apical membrane (hemidesmosome-like structures) of tendon cells was evident. On the basal side the microtubules were positioned close to the electron dense plaques along the basal membrane, engaged in myotendinous junction.

**Figure 3.**
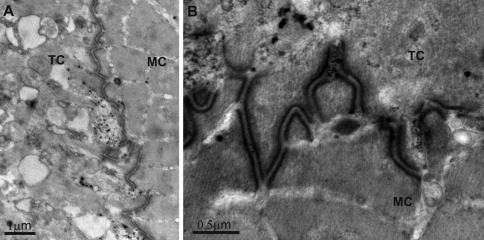
Myotendinous junction in the dorsal parts of pereonites of premolt adult *Ligia italica* is an extensive anchoring junction. The entire zigzag folded basal and apical membranes of tendon and muscle cells, respectively, are engaged in this outstanding intercellular mechanical connection **(3A)**. Prominent electron dense cytoplasmic plaques are evident along both cell membranes, separated by a thin layer of extracellular matrix **(3B)**. **MC** muscle cell **TC** tendon cell.

Prominent anchoring junctions were evident between muscle cells and tendon cells (Fig. 3). The entire basal membrane of the tendon cell was intensely folded in a zigzag pattern, exactly matching the folding of the muscle cell sarcolemma beneath. Both cell surfaces contributing to this junction were closely apposed and the intervening layer of the extracellular matrix material was not conspicous. The complex connection between these two cells in premolt specimens corresponds to characteristic design in animals with one complete cuticle (Supplementary figure), comprising electron dense plaques beneath both cell membranes and extracellular material in the narrow intercellular space.

### Intramarsupial premolt animals

**Figure 4.**
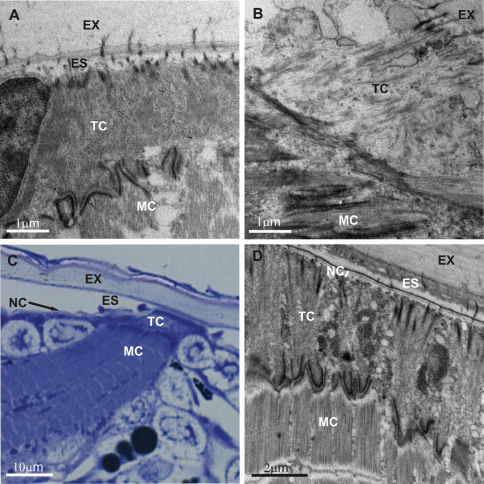
Overview of exoskeleton anchoring to tendon cells and muscles in intramarsupial developmental stages of *Porcellio scaber*. In prehatching embryos **(4A, B)** and in premolt marsupial mancas (**4C** a semithin section and **4D**) the connections between the exoskeleton, tendon cells and muscle cells were already established. **EX** exoskeleton **ES** ecdysal space **NC** new cuticle **TC** tendon cell **MC** muscle cell.

Development of *Porcellio scaber* embryos and marsupial mancas involves renewal of the exoskeleton. Prehatching embryo and several marsupial mancas displaying morphological attributes of premolt were analysed in this study. The detachment of the old cuticle and disintegration of the basal parts of the detached cuticle were identified in the integument of these specimens. In addition, the substantial new cuticle formation was evident in marsupial mancas. The exoskeleton in the prehatching embryo and in the premolt marsupial mancas was extensively connected to tendon cells and muscles underneath and extensive intercellular junctions between muscle cells and tendon cells were established (Fig. 4).

**Figure 5.**
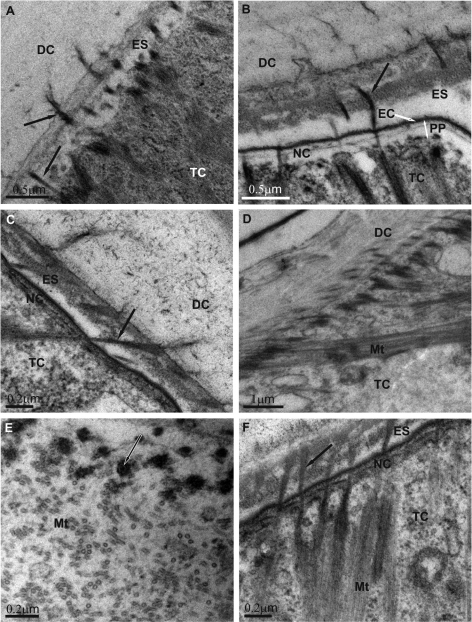
Anchoring junctions between the detached cuticle and the apical part of tendon cells in the prehatching embryo **(5A, D, E)** and marsupial mancas **(5B, C, F)** of *Porcellio scaber* (ultrathin cross-sections). The newly forming cuticle in marsupial mancas, consisting of the epicuticle and pre-ecdysal procuticle, was mechanically connected to tendon cells. Numerous bundles of fibers (arrows) running from tendon cells through the new cuticle and ecdysal space into the detached cuticle are evident. Microtubules were found in close proximity to electron dense plaques at the apical surface of tendon cells. **DC** detached cuticle **NC** new cuticle **EC** epicuticle **ES** ecdysal space **PP** pre-ecdysal procuticle **TC** tendon cell **Mt** microtubules.

Numerous fibrous connections between the detached cuticle and the apical membrane of tendon cells were evident in all premolt intramarsupial animals examined (Fig. 5). The newly forming cuticle in marsupial mancas, consisting of epicuticle and a few layers of the pre-ecdysal procuticle, was mechanically connected to tendon cells.

**Figure 6.**
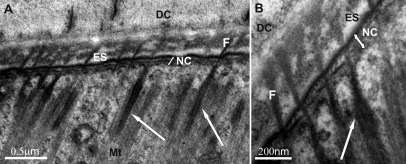
Electron dense plaques (hemidesmosome-like structures) at the apical surface of the tendon cells in the marsupial manca of *Porcellio scaber*. Electron dense plaques (arrows) are associated with microtubules **(Mt)** in the cytoplasm of the tendon cell and with the bundles of fibers **(F)** running through the ecdysal space **(ES)** on the opposite side. **DC** detached cuticle **NC** new cuticle.

**Figure 7.**
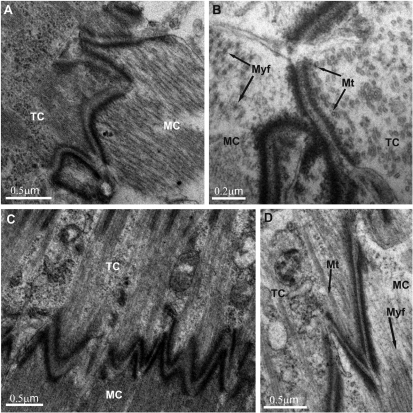
Myotendinous junction in the *Porcellio scaber* prehatching embryo **(7A, B)** and marsupial mancas **(7C, D)**. The cytoplasmic plaque of the anchoring junction at the basal membrane of tendon cell is more lucent and thinner than the accompanying plaque at the apical membrane of the muscle cell in the prehatching embryo **(7A, B)**. Microtubules of the tendon cells are in close proximity to the basal dense plaques. **TC** tendon cell **MC** muscle cell **Mt** microtubules **Myf** myofilaments.

Tendon cells ultrastructurally resembled the adult arthropod tendon cells and were characterized by apicobasal arrays of microtubules. Apically, the microtubules were found close to oblong electron dense regions (hemidesmosome-like structures), aligned in the same direction (Figs 5D–F and Fig. 6). In longitudinal and in oblique sections the profiles of microtubules were evidenced in close proximity to the electron dense plaques of junctions along the tendon cell basal membrane in both, prehatching embryos (Figs 7A, B) and marsupial mancas (Figs 7C, D).

Myotendinous junctions displayed a characteristic zigzag outline, occupying the entire tendon – muscle interface (Fig. 7). From the structural point of view, the myotendinous junctions in marsupial mancas closely resembled these junctions in adult arthropods, comprising a thin layer of extracellular material between cell membranes and dense cytoplasmic plaques of approximately equal densities and thicknesses below the both cell membranes (Figs 7C, D). On the other hand, in the prehatching embryo, the intracellular membrane-associated layer of dense cytoplasmic material contributing to the anchoring junction in a tendon cell was more lucent and thinner than that contributing to the junction in a muscle cell (Figs 7A, B).

## Discussion

Specialized anchoring complexes between exoskeleton, tendon cells and force-generating muscle cells are essential features of the musculoskeletal system in arthropods (Noble-Nesbitt 1963, Mellon 1992, Lai-Fook and Beaton 1998, Bitsch and Bitsch 2002). A complex network of cytoskeletal and junctional elements constitutes the main scaffold of specialized mechanical connections between the calcified exoskeleton and underlying tissues in crustaceans (Mellon 1992). These elaborate connections between different types of cells and hierarchically structured extracellular matrix constitute a common functional network, enabling animal movements, and are thus involved also in animal responses to various physiological and environmental cues.

Our results show that the old exoskeleton is still mechanically attached to the underlying epithelium in the regions of muscle attachment sites for a certain period of the premolt phase in isopod crustaceans. We have observed massive arrays of fibers running from tendon cells through the new cuticle and ecdysal space up to the distal layers of the detached cuticle in adult premolt *Ligia italica* and in premolt intramarsupial specimens of *Porcellio scaber*. The continuity of these fibers was clearly evidenced in both species. The fibers extending from the tendon cells deep into the cuticular matrix in non-molting specimens are known from previous studies of arthropods (Noble-Nesbitt 1963, Mellon 1992, Lai-Fook and Beaton 1998) and were also reported in some studies of molting insect species (Lai-Fook 1967, Caveney 1969, Lai-Fook and Beaton 1998) and in two studies of molting crustaceans (Buchholz and Buchholz 1989, Yamada and Keyser 2009).Different authors use different names to designate these fibers, which is rather confusing. They are termed muscle attachment fibers (Caveney 1969), tonofibrillae (Buchholz and Buchholz 1989, Lai-Fook and Beaton 1998, Tucker et al. 2004), tonofilaments (Subramanian et al. 2003), intracuticular fibers (Mellon 1992) and intracuticular rods (Criel et al. 2005). To the best of our knowledge, the macromolecules constituting these fibers have not been identified yet, neither in insects, nor in crustaceans. They are described as dense strands of cuticular material that project into the apical invaginations of tendon cells (Tucker et al. 2004). In contrast to comprehensively resolved molecular architecture of the tendon – muscle junction in *Drosophila* (Volk 2006, Schweitzer et al. 2010), the proteins that connect the apical surface of the epidermis to the cuticle are not known (Brown 2000). The ultrastructural organization of the fibrous connections between the detached cuticle and tendon cells described in our study for two isopod species closely resembles that reported for the premolt *Euphausia superba* by Buchholz and Buchholz (1989) and for the premolt podocopid ostracods by Yamada and Keyser (2009). As in *Euphausia superba* and in ostracods the new preecdysal cuticle in isopods is secreted while fibers maintain the connection between the old cuticle and epidermis. Yamada and Keyser (2009) in addition described the formation of the new cuticle in detail. They reported that the new epicuticle was deposited around the extended intracuticular fibers and suggested that the intracuticular fibers increase their length before the deposition of the new epicuticular material. The continuity of the fibers stretching from the tendon cells through the new cuticle and ecdysal space to the old cuticle is clearly evident in our images of premolt isopod integument. This indicates that the structural reorganization of the existing fibers occurs during premolt rather than extensive formation of the new fibers. This is also in agreement with previous reports on the continuity of the tonobrillae in molting insects (Lai-Fook 1967) and crustaceans (Buchholz and Buchholz 1989, Yamada and Keyser 2009). We consider that cuticle-muscle attachment complexes supposedly helping or enabling movements during shedding of the old cuticle are also involved in maintenance of integument integrity of the 'two-cuticle' stage in the premolt isopods. Fibers connecting the detached cuticle to the underlying tissues likely serve as final anchoring sites before exuviation. These anchoring connections to the underlying epidermis and muscles may also be involved in limited locomotory activity of the animal during molting. As the newly forming cuticle underneath is already extensively connected by such fibers to the apical membrane of tendon cells and indirectly to muscles below, at least elementary locomotion may thus be enabled also immediately after exuviation.

Apicobasally oriented microtubule arrays are formed in several types of polarized epithelial cells. Tucker et al. (2004) report that most microtubules in *Drosophila* tendon cells exhibit unusually large diameters of up to 30 nm, while in our study the usual microtubules of 24 nm in diameter were observed in the tendon cells of adult and intramarsupial isopods. Here we show that tendon cells in prehatching embryos and marsupial mancas of *Porcellio scaber* already include a substantial apicobasally oriented transcellular arrays of microtubules, evidently engaged in myotendinous junctions and in apical anchoring of the cuticular matrix. Thus we consider that in the prehatching embryos and marsupial mancas the structural framework of musculoskeletal linkage is basically established and could assist in animal motions within the marsupium. This consideration is further supported by our observation that marsupial mancas display pronouced body movements inside the marsupial fluid and is supported also by the study of Milatovič et al. (2010), who reported that embryo hatching from the vitelline membrane involves swelling and active movement.

The myotendinous junction in the dorsal parts of pereonites in molting *Ligia italica* is a zigzag patterned junction of the tendon cell basal membrane and muscle cell sarcolemma, with an inconspicious layer of extracellular matrix inbetween. The entire basal surface and apical surface of tendon and muscle cell, respectively, contribute to this heterotypic adherens junction. Both interacting cell membranes are extensively folded, which increases the surface area of contact and contributes to enhanced mechanical resistance. The myotendinous connection in molting *Ligia italica* structurally resembles that described in non-molting specimens and in *Drosophila*. The myotendinous junction in *Drosophila* is considered to be composed of two sets of hemiadherens junctions with an intervening layer of extracellular matrix material that has a substantial thickness in certain situations (Prokop et al. 1998, Tucker et al. 2004). In recent years the molecular machinery involved in the formation of these junctions has been increasingly elucidated. The *Drosophila* myotendinous connection is integrin dependant and involves transmembrane integrins that connect to protein ligands in the extracellular matrix and to the cytoskeleton inside the cell (Prokop et al. 1998, Delon and Brown 2008). This strategy is implemented in both, direct and indirect muscle attachments in *Drosophila*. The molecular composition of tendon cell – muscle cell junction has not been resolved in crustaceans, but similar principles involving integrins and associated linking proteins are expected to be implied.

The ultrastructural arhitecture of myotendinous junctions in the prehatching embryos and marsupial mancas of *Porcellio scaber* analysed in this study is similar to the general structural outline of adult arthropod muscle attachments. In marsupial mancas it appears to be structurally fully elaborated, while in the prehatching embryo it may not be completely formed. The cytoplasmic plaques engaged in anchoring junctions at the basal membrane of the tendon cell in the prehatching embryo are markedly electron lucent and thinner as compared to the opposing cytoplasmic plaques in the muscle cells, while in adult arthropods the myotendinous anchoring junctions comprise cytoplasmic plaques of similar thicknesses and densities in both cells. A similar situation was observed in *Drosophila* in vitro culture of primary embryonic cells by Tucker et al. (2004), who reported that differentiating tendon cells are characterized also by thinner hemiadherens junction as compared to the associated muscle cell. Thus we consider that this structural feature may indicate the not completely differentiated junction. The functional significance of this is not yet clear.

## Conclusions

Cell to cell and cell to matrix anchoring junctions, together with their associated cytoskeletal elements, are engaged in providing tissue structural scaffold and integrity, but more than that, they are increasingly discussed from the perspective of tissue and cell dynamics (Zaidel-Bar et al. 2010). These junctions undergo dynamic changes during development and during regeneration in adulthood. The overall general architecture of the exoskeleton-muscle attachment in isopod crustaceans described in our study is similar to that reported for other arthropods.We show here that elaborate anchoring junctions between the tendon cell apical membrane and the extracellular matrix provide attachment of the exoskeleton to the underlying tissues also during cuticle replacement in adult and in intramarsupial developmental stages of isopod crustaceans. Thus they contribute to integument integrity during molting and together with associated microtubules in tendon cells and myotendinous connections likely enable at least basic movements in this period. As cuticle replacement involves old cuticle detachment followed by the new chitin – protein matrix secretion at the apical membranes of epidermal cells, the rearrangement and remodeling of anchoring junctions between the tendon cells and cuticle are expected. The continuity of the fibers ranging from the tendon cells through the new cuticle and ecdysal space to the old cuticle was evident in premolt adult and intramarsupial isopods. This indicates that during premolt the reorganization of fibers and their associations with the cuticle takes place, rather then extensive formation of the new fibers. Our considerations are in agreement with previous reports on the continuity of tonofibrillae, which maintain the connection between the tendon cells and the old cuticle in molting insects (Lai-Fook 1967) and crustaceans (Buchholz and Buchholz 1989, Yamada and Keyser 2009).

## Supplementary figure

Exoskeleton-muscle attachment in the adult Ligia italica with the entire cuticle of the usual thickness. (doi: 10.3897/zookeys.176.2445.app) File format: Portable (Public) Network Graphic (png).
